# Stem retention and survival in revision of anatomical convertible shoulder arthroplasty to reverse arthroplasty: a Dutch registry study

**DOI:** 10.1186/s12891-021-04247-z

**Published:** 2021-04-28

**Authors:** Luuk M. A. Theelen, Ben Mory, Sharmila Venkatesan, Anneke Spekenbrink-Spooren, Loes Janssen, Frederik O. Lambers Heerspink

**Affiliations:** 1 Department of Orthopaedic Surgery, VieCuri Medical Center, Venlo, The Netherlands; 2 Dutch Arthroplasty Register (Landelijke Registratie Orthopedische Implantaten), ‘s Hertogenbosch, The Netherlands

**Keywords:** Stem retention, Survival, Revision, Anatomical convertible shoulder arthroplasty

## Abstract

**Background:**

Convertible stem designs allow for stem retention during revision from anatomical to reverse shoulder arthroplasty. In some cases conversion is not possible for example due to excessive soft tissue tensioning. In these cases a total revision is necessary. The primary aim of this Dutch registry study was to evaluate the unforeseen stem reversion percentages in revision of convertible anatomical shoulder arthroplasty to reverse shoulder arthroplasty.

**Methods:**

Shoulder arthroplasties (*n* = 2834) performed between 2014 and 2016 registered in the Dutch Arthroplasty Registry were selected. In 2016 94% of primary arthroplasties and 92% of revision arthroplasties were registered in the database. Arthroplasties were selected on convertibility. Mean follow-up was 2.4 years. We analysed the number of revisions for convertible and non-convertible designs. Cases with obligatory revisions as periprosthetic joint infections, stem loosening and periprosthetic fractures were excluded. Kaplan-Meier analysis was used to calculate humeral stem survival. Multivariate cox-regression analysis was used to determine risk factors for stem revision.

**Results:**

The majority of procedures (respectively 90.9 and 72.1% for the convertible and non-convertible group) concerned a conversion to reverse shoulder arthroplasty (*p* = .02). In the convertible group, the stem was retained in 29 out of 40 patients (72.5%). Overall implant survival was 94.5% after a mean follow-up of 2.4 years. Hemiartroplasty, fracture as primary indication, previous shoulder surgery and lower age were risk factors for revision.

**Conclusions:**

Although convertible designs are gaining popularity due to their expected advantage in revision arthroplasty, surgeons should be aware that during a revision procedure in 27.5% of the patients an unforeseen stem revision is necessary.

**Supplementary Information:**

The online version contains supplementary material available at 10.1186/s12891-021-04247-z.

## Background

The number of shoulder arthroplasties performed has been steadily rising over the past decades [1–6]. With an increase in the number of primary arthroplasties, the number of revision procedures are rising as well. The most common reasons for revision of anatomical shoulder arthroplasties are periprosthetic joint infection, instability/dislocation, secondary rotator cuff insufficiency, or aseptic loosening [7]. In the past, conversion of an anatomical total shoulder arthroplasty to a reverse shoulder arthroplasty required removal of the stem. In 20–25% of the cases this is associated with a risk of humeral fractures, or a need for osteotomy of the humerus to remove a fixed cemented stem. This could result in extensive soft tissue damage [3, 6, 8–12]. Convertible stem systems were designed to retain the humeral stem and convert it to a reverse prosthesis more easily. This is claimed to result in a lower complication rate, shorter operation time, less blood loss and fewer reinterventions [6, 11, 13–19]. A well-known problem in these conversions is excessive soft tissue tensioning, therefore the shoulder stem cannot be retained, or it impairs range of motion, causing an unforeseen revision. This in contrast to foreseen revision in the case of loosening, periprosthetic fractures and infections. The literature describes a stem revison percentage ranging between 0.0 and 62.5% [9, 12, 16, 18, 19]. However, most studies regarding this subject comprise small cohorts.

The primary goal of our study was to evaluate the unforeseen revision rate in modular stems in revision procedures from anatomical (total or hemi) to reverse shoulder arthroplasty in The Netherlands. Based on previous studies we hypothesized that a considerable number of convertible stems inserted in primary shoulder arthroplasty are not retained during revision arthroplasty, due to unforeseen circumstances. Secondly, we compared overall survival rates of the stem between shoulder arthroplasties (both the complete prosthesis and the stem separately) comprising a modular stem, and those comprising a non-convertible stem. Thirdly, we aimed to identify factors associated with stem revision.

## Methods

For this cohort study, we retrospectively reviewed the Dutch Arthroplasty Register (LROI) database containing all shoulder arthroplasties performed between January 2014 and December 2016. The LROI is a nationwide registry for joint prostheses established by the Dutch Orthopaedic Association (NOV) in 2007 with over 100 affiliated institutes. Registration of shoulder arthroplasties started in January 2014. In 2016 94% of primary arthroplasties and 92% of revision arthroplasties were registered in the database.

### Population

We identified a total of 2834 registered primary arthroplasties. The registry supplied a list of types and brands of shoulder prostheses, which were labelled either as convertible or non-convertible by two researchers (LT, OLH) (Supplemental file).

**Fig. 1:**
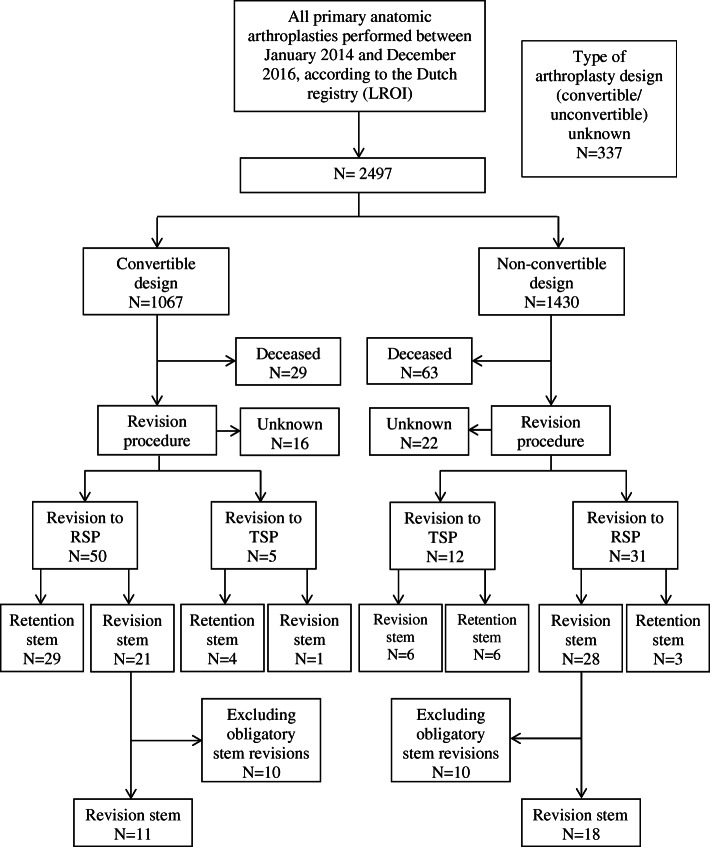
Study design

For some non-convertible designed prostheses an adaptor is developed to enable revision to reversed without the need for a stem revision. As these designs were not primarily designed for conversion to reverse shoulder arthroplasty they were defined as non-convertible (*n* = 3). Stemless designs were excluded from this study. After exclusion of patients with unknown stem type due to missing data (*n* = 337), patients were divided into two cohorts: a convertible group (*n* = 1067) and a non-convertible group (*n* = 1430). Follow up was at least 1 year. The study flow is presented in Fig. 1.

### Data collection

The registration included demographic patient information, diagnostic information, and procedural information about both the primary as well as, when applicable, the revision procedures. The registry provided all the data anonymously, therefore if the type of the revision procedure was unknown (revision to total, reversed shoulder arthroplasty or extraction) these patients were excluded.

#### Outcome measures

The primary outcome of this study was to evaluate the unforeseen revision rate of convertible stems used in revision of anatomical (total and hemi) shoulder arthroplasty to reversed shoulder arthroplasty. In some cases, stem revision can be foreseen preoperatively, i.e. prosthetic joint infection, stem loosening and periprosthetic fractures. This was done for purpose of comparison to the literature and to provide surgeons insight in the amount of unforeseen stem-revision in clinical practice [3, 9, 18–20]. A secondary analysis was performed to determine the overall stem revision rate including foreseen stem revisions. Secondary outcomes were: overall prosthesis and stem survival in the convertible and the non-convertible design.

### Statistics

Statistical analysis was performed using SPSS (IBM SPSS Statistics for Windows, version 24.0 [IBM Corp., Armonk, N.Y., USA]). Descriptive statistics (mean, median, range) were applied to describe patient characteristics. Categorical variables were analysed using Chi-square tests, while continuous variables were analysed depending on their distribution. In case of a normal distribution, the two-sample independent T-test was used. In case of continuous variables without a normal distribution or ordinal variables the Mann-Whitney test was used. Survival of the entire prosthesis, as well as survival of the humeral stem separately, were analysed by means of Kaplan-Meier analyses including the log rank test to compare convertible and non-convertible prostheses. Cox-regression survival analysis was used to calculate hazard ratios for the risk of stem revision and to correct for possible confounders. Independently, variables with a *p*-value < 0.1 in a univariate Cox regression analysis were included in the multivariate analysis.

A *p*-value < 0.05 was considered statistical significant. Missing values were not replaced.

## Results

### Demographics

Demographic patient characteristics are presented in Table 1. The total cohort consisted of 2834 primary anatomic (total and hemi) shoulder arthroplasties (Fig. 1). A total of 2009 women (70.9%) and 818 men (28.9%) were included. Information on gender was missing in 7 patients (0.2%). At the time of the primary procedure, the mean age was 66.3 years (SD 10.8) and mean follow-up was 2.4 years (SD 0.9). Overall implant survival was 94.5%.

**Table 1:** Patient demographics of both the convertible stem group as well as the non-convertible stem group (SD: Standard deviation; ASA: American Society of Anesthesiologists classification)

	Convertible stems (***n*** = 1.067)	Non-convertible stems (***n*** = 1.430)	***P***-value
**Age (years)**, Mean (SD)	66.4 (10.5)	66.9 (10.7)	0.33
**BMI (kg/m**^**2**^**),** Mean, SD	28.7 (5.5)	28.6 (5.7)	0.79
**Sex**, Male ()	26.9	28.5	0.37
**Smoker**, Yes (%)	17.1	14.5	0.08
**ASA** (%)			0.05
I	11.9	14.5	
II	67.6	63.1	
III-IV	20.5	22.4	
**Walch classification** (%)			0.61
A1/A2	80.7	79.6	
B1	13.7	13.9	
B2/B3	5.5	6.5	
**Percentage/year** (%)			**< 0.001**
2014	28.6	71.4	
2015	45.1	54.9	
2016	53.7	46.3	
**Side** (%)			0.79
Left	47.1	47.7	
Right	52.9	52.3	
**Diagnosis** (%)			
Osteoarthritis	67.5	77.3	**< 0.001**
Fracture	25.8	20.2	
Other	6.8	2.4	
**Approach** (%)			**< 0.001**
Deltopectoral	88.2	93.6	
Anterosuperior	11.8	6.4	
**Prosthesis type** (%)			0.36
Hemi	37.6	39.4	
Total	62.4	60.6	
**Fixation** (%)			**< 0.001**
Cemented	20.0	37.9	
Uncemented	80.0	62.1	
**Previous surgery,** No (%)	86.8	88.0	0.37

The convertible group consisted of 1067 patients with a mean age of 66.5 (SD 10.4) and the non-convertible group consisted of 1430 patients with a mean age of 66.9 (SD 10.7) at the time of the primary arthroplasty (*p* = .39). Mean follow-up for the convertible design prostheses was 2.1 years (SD 0.84) and 2.5 years (SD 0.91) for the non-convertible prostheses (*p* < 0.01). Between 2014 and 2016 an increase of 28.6 to 53.7% was observed in the use of convertible stem designs (*p* < 0.01).

In 38 patients the type of revision was unknown and were therefore excluded from analysis. The distribution of anatomic total shoulder arthroplasties and hemiarthroplasties used in the primary procedure did not differ significantly between the convertible (total: 62.4%; hemi: 37.6%) and the non-convertible group (total: 60.6%; hemi: 39.4%) (*p* = 0.40). The initial diagnosis for the arthroplasty procedure was osteoarthritis in the majority of patients in both groups: 67.5% versus 77.3% in the convertible and non-convertible group, respectively, followed by fractures (25.8% vs 20.2%). Significant differences were found in surgical approach (deltopectoral vs. anterosuperior) with more anterosuperior approach in the convertible group (11.4 vs. 6.7%) (*p* < 0.01). Also, type of fixation (cemented vs. uncemented) varied significantly in both groups. In the convertible group 80% of the stems were uncemented, compared to 62.1% in the non-convertible group (*p* < 0.01).

#### Primary outcome

##### Unforeseen stem revision to reversed shoulder prosthesis

In the convertible group, in 16 patients it was unknown if the revision was to a reverse or anatomical type arthroplasty or a extraction was performed. Fifty revisions concerned conversion to a reversed shoulder prostheses. In comparison, within the non-convertible group in 22 patients the type of revision surgery was unknown. Thirty-one revisions concerned conversion to a reversed shoulder prostheses. In the convertible group, the stem was retained in 29 out of 50 patients (58.0%).

Ten cases in the convertible group were marked as obligatory stem revisions (cause of revision: stem loosening, periprosthetic infection). Excluding the foreseen revisions, the stem was retained in 29 of 40 revision procedures, leading to a stem retention percentage of 72.5%.

#### Secondary outcomes

##### Overall prosthesis survival

**Fig. 2:**
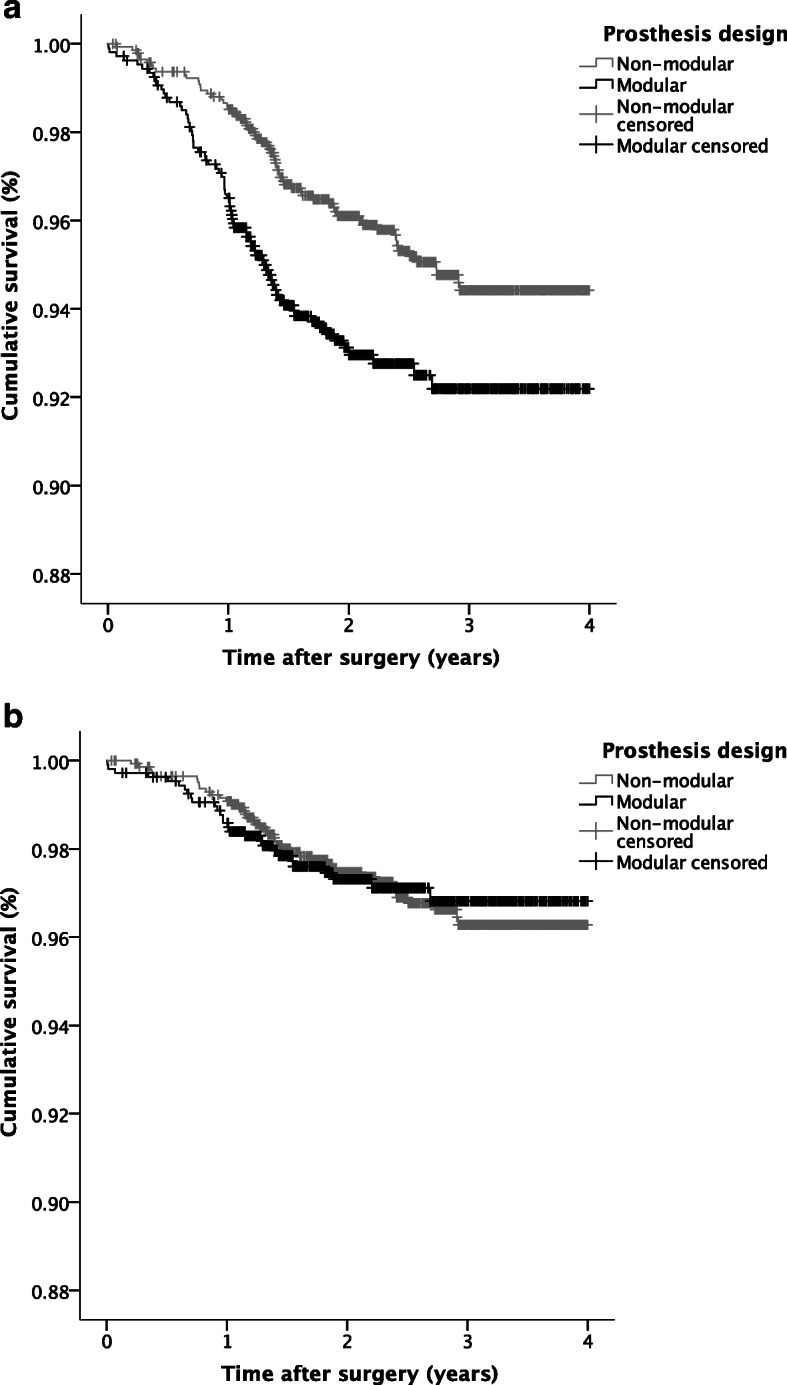
Kaplan Meier Survival plots comparing survival in the convertible vs the non-convertible group. Plot (**a**) depicts survival of the entire prosthesis, whereas plot (**b**) depicts survival of the humeral stem

Seventy-one of the 1067 (6.7%) convertible prostheses placed, required a revision procedure compared to 65 of 1430 (4.5%) of the non-convertible prostheses (*p* = .02) (Fig. 1). The higher revision rate for convertible prostheses was confirmed by the Kaplan Meier analysis, showing lower survival for convertible prostheses compared to non-convertible prostheses (*p* < 0.01) (Fig. 2a). However, after correction for confounders (age, smoking status, Walch classification, diagnosis, prosthesis type, previous surgery), the difference in overall prosthesis survival between convertible and non-convertible prostheses disappeared (HR 1.36; 95% CI 0.93–2.00). A lower age, a fracture as primary indication for surgery, a hemi shoulder arthroplasty and previous shoulder surgery all independently increased the risk of revision (*p* < 0.05) (Table 2).

**Table 2:** Univariate and multivariate cox regression analyses for overall prosthesis survival and for survival of the stem separately. (HR: hazard ratio; ASA: American Society of Anesthesiologists classification)

	Outcome: Prosthesis survival (overall)				Outcome: Survival of the stem			
	Crude HR (95% CI)	***p***-value	Adjusted HR (95% CI)	***p***- value	Crude HR (95% CI)	***p***-value	Adjusted HR (95% CI)	***p***-value
**Convertible stem**								
*No*	1		**1**		1		1	
*Yes*	1.6 (1.2–2.3)	**< 0.01**	1.4 (0.9–2)	0.1	1 (0.6–1.6)	0.9	0.8 (0.5–1.5)	0.5
**Age**	0.97 (0.95–0.98)	**< 0.01**	0.97 (0.95–0.99)	**< 0.01**	0.97 (0.95–0.99)	**< 0.01**	0.99 (0.96–1.01)	0.2
**BMI**	1 (0.97–1.03)	0.9			1 (0.96–1.04)	0.97		
**Sex**								
*Male*	1				1			
*Female*	0.9 (0.6–1.3)	0.5			0.7 (0.4–1.1)	0.1		
**Smoking**								
*No*	1		1		1			
*Yes*	1.6 (1–2.4)	**0.04**	1 (0.6–1.6)	0.9	1.6 (0.9–2.9)	0.1		
**ASA**								
*I*	1				1			
*II*	1 (0.6–1.6)	0.8			0.9 (0.5–1.8)	0.8		
*III-IV*	1 (0.6–1.8)	1			0.6 (0.3–1.5)	0.3		
**Walch classification**								
*A1/A2*	1		1		1			
*B1*	0.4 (0.2–0.9)	0.02	0.6 (0.3–1.2)	0.2	0.6 (0.2–1.5)	0.3		
*B2/B3*	0.7 (0.3–1.6)	0.4	0.7 (0.3–2)	0.5	0.9 (0.3–2.7)	0.8		
**Year**								
*2014*	1				1			
*2015*	0.9 (0.6–1.3)	0.5			0.8 (0.5–1.4)	0.5		
*2016*	1.1 (0.7–1.7)	0.6			1.1 (0.6–2)	0.8		
**Side**								
*Left*	1				1			
*Right*	0.8 (0.6–1.1)	0.2			0.7 (0.4–1.1)	0.7		
**Diagnosis**								
*Osteoarthritis*	1		1		1		1	
*Fracture*	2.9 (2.0–4.1)	**< 0.01**	1.8 (1.1–2.8)	**0.01**	2.1 (1.3–3.5)	**0.02**	1.5 (0.9–2.7)	0.1
*Cuff arthropathy*	0.9 (0.3–2.9)	0.9	1.3 (0.4–4.1)	0.7	0.5 (0.07–3.7)	0.5	0.7 (0.1–5.5	0.8
*Other*	2.4 (0.6–9.7)	0.2	1.7 (0.4–7.0)	0.5	2 (0.3–14.4)	0.5	1.6 (0.2–11.8)	0.6
**Approach**								
*Deltopectoral*	1				1			
*Anterosuperior*	0.9 (0.5–1.6)	0.7			0.8 (0.3–1.9)	0.6		
**Prosthesis**								
*Total shoulder prosthesis*	1		1		1		1	
*Hemi shoulder prosthesis*	2.5 (1.8–3.5)	**< 0.01**	2 (1.3–3.1)	**< 0.01**	2.2 (1.4–3.6)	**< 0.01**	2 (1.2–3.4)	**0.01**
**Fixation**								
*Uncemented*	1				1			
*Cemented*	1.1 (0.8–1.6)	0.5			0.8 (0.5–1.4)	0.5		
***Previous surgery***								
*No*	1		1		1		1	
*Yes*	2.7 (1.8–4)	**< 0.01**	1.9 (1.2–3)	**< 0.01**	4.3 (2.6–7.1)	**< 0.01**	3.9 (2.3–6.6)	**< 0.01**

### Overall survival of the stem

The results of overall survival analyses of the stem revealed that in 28 of the 1067 (2.6%) convertible arthroplasties placed a revision of the humeral stem was required, versus 43 of the 1430 (3.0%) of the non-convertible prostheses (*p* = 0.6). In the non-convertible group revision of the stem was not required in case of: glenoid revision, usage of an adapter or humeral head revision. The corresponding Kaplan-Meier survival graph is shown in Fig. 2b. Due to the crossing survival curves a log rank test was not indicated. After correction for possible confounders, survival of the humeral stem remained similar for convertible vs non-convertible designs (HR 0.8; 95% CI 0.5–1.5).

Risk factors for survival of the entire prosthesis as well as survival of the humeral stem, are depicted in Table 2. A hemi shoulder arthroplasty and a previous shoulder surgery were both found to independently increase the risk of humeral stem revision (HR 2; 95% CI 1.2–3.4 and HR 3.8; 95% CI 2.3–6.6, respectively).

Outliers regarding revisions of certain brands of arthroplasties were not identified. In the convertible group 13 subtypes of prostheses were revised in 28 cases and in the non-convertible group 23 subtypes were revised in 43 revisions.

## Discussion

The primary goal of this study was to evaluate the revision rate of convertible stems used in revision of anatomical (total and hemi) shoulder arthroplasty to reversed shoulder arthroplasty. The amount of unforeseen stem-revision (excluding the periprosthetic joint infections and stem loosening) in these revisions from anatomic/hemi to reverse was 27.5%. In total, including the obligatory stem revisions the stem was revised in 42.0%. After correction for confounders no significant differences were observed in overall prosthesis survival and stem survival between both groups. Overall implant survival was 94.5% after a mean follow-up of 2.4 years. No outliers were found regarding revision of certain brands of prostheses.

In our registry study in 27.5% an unforeseen stem revision was performed, in 72.5% of the revision to reversed procedures the convertible stem was retained. Previous studies on the topic of convertible humeral stem revisions revealed comparable stem retention rates ranging from 65.3–78.0% [3, 9, 18–20]. In these studies patients with periprosthetic joint infections and stem loosening were excluded as in these indications stem revision is obligatory. Dilisio et al. reported a retention rate of 37.5%, however revisions such as periprosthetic joint infections, loosening were included in this series, explaining the lower stem retention rate compared with other studies [16].

Due to the fact that we received anonymous registry data, case specific information regarding why the stem was revised was missing and could not be retrieved. A too minimal humeral head resection in the primary procedure may lead to a too proud position of the humeral stem which can put the cuff under stress and can result in cuff failure. Revision of these cases with malpositioning of the stem are at high risk of excessive tissue tensioning. Most common reasons for stem revision in the convertible group in literature are excessive tissue tensioning that makes the reduction of the reverse shoulder arthroplasty impossible or causes limited range of motion [6, 10, 19]. Surgeons should be aware that not all revisions in the convertible group are simple revisions. Excluding patients with stem loosening and periprosthetic joint infections in 27.5%% of patients still a stem revision needs to be performed. One should be prepared to perform total revision arthroplasty.

After correcting for confounders we found no significant difference in survival rate between both groups. However, we found a significant rise in the use of convertible stem designs. A possible explanation for this finding is that revision surgery in convertible shoulder arthroplasty yields less complications and is less time consuming [6, 12–19].

Despite this advantage we did not find an increased revision percentage of the convertible stems compared to the non-convertible stems. However, in the convertible group 9% (5 / 55) of anatomical or hemi arthroplasties were revisions to a total or hemi shoulder arthroplasty, as in the non-convertible group this was the case in 28% (12/43). This might be a reflection of the greater morbidity required to remove a well fixed stem. As this is a registry study we don’t have case specific information to confirm the hypothesis.

The overall survival reported in the LROI was 94.6%. The New Zealand registry showed a 2-year survival of 96.6% [21]. Five-year survival percentages vary between 94.4–98.9% [21–23]. In our study, the convertible group consisted of 37.6% hemiarthroplasties vs 39.4% in the non-convertible group. Our findings have shown that previous shoulder surgery, fracture as indication, lower age and hemiarthroplasty were risk factors for revision after multivariate cox regression analysis. Consequently, the high percentage of hemiarthroplasties in our cohort might explain the higher revision rate compared to literature.

A major strength of this study was that the cohort included a large number of patients. By using the LROI, a nationwide orthopaedic implantation registry, we created a large study group treated in all centres throughout The Netherlands. Furthermore, the LROI reported a completeness of data collection of 94% of primary procedures and 92% of revision procedures.

The LROI started collecting data of shoulder arthroplasties in 2014, which made the mean follow-up time of 2.4 years relatively short. Sixteen patients were excluded from the stem retention rate analysis due to missing data. In these patients it was not clear if they were revised to anatomical/ hemi, reverse shoulder arthroplasty or an extraction was performed. This may have resulted in a selection bias leading to an over or under estimation of the actual retention rate. As the data was anonymized the reason and type of revision could not be determined.

As part of a registry study the findings are based on a heterogenic group of patients, treated by many different surgeons, using many different designs for several indications. As a consequence, our findings are representative for the population as a whole, but are less applicable to specific designs.

## Conclusion

Recently, there has been a profound rise in use of convertible shoulder arthroplasty among Dutch shoulder surgeons. It is assumed that revision of a convertible stem is less time consuming and yields less complications compared to removal of a well fixed stem. In approximately 72.5% of the unforeseen revision from anatomical / hemi to reversed arthroplasty the convertible stem could be retained. Surgeons and patients should be well informed that in 27.5% of patients the stem need to be revised.

## Supplementary Information

**Additional file 1.** Classification of convertible and non-convertible prostheses.

## Data Availability

The datasets used and/or analysed during the current study are available from the corresponding author on reasonable request.

## Abbreviations

HR: Hazard ratio
LROI: Dutch Arthroplasty Register
NOV: Dutch Orthopaedic Association
SD: Standard deviation
