# Supplementary material for: Stem retention and survival in revision of anatomical convertible shoulder arthroplasty to reverse arthroplasty: a Dutch registry study
Source: BMC Musculoskelet Disord. 2021 Apr 28;22:396. doi: 10.1186/s12891-021-04247-z (PMC8082907; doi:10.1186/s12891-021-04247-z)
Supplement: Supplementary file 1 — Additional file 1. Classification of convertible and non-convertible prostheses. [file 12891_2021_4247_MOESM1_ESM.docx]

| **Modular Stem Design** | **Nonmodular Stem Design** |
| --- | --- |
| Aequalis Ascend Flex | Aequalis Ascend monolithic |
| Affinis Fracture | Aequalis Fracture hemiarthroplasy |
| DePuy Synthes Global Unite | Aequalis Press-fit |
| Exactech Equinoxe | Aequalis Primary |
| FX Solutions Humelock II | Aequalis Reverse |
| FX Solutions Humeris | Aequalis Reverse fracture |
| Johnson Delta X-tend modular humeral stem | Affinis Inverse |
| Lima SMR | Affinis Short |
| Lima SMR stemless | Affinis Total shoulder |
| ZimmerBiomet Anatomical Fracture stem | Arthrex Eclipse |
| ZimmerBiomet Anatomical Shoulder stem | Arthrex Univers II |
| ZimmerBiomet Comprehensive | Arthrex Universe Fracture |
| ZimmerBiomet TM reverse | DePuy Synthes Global FX |
|  | DePuy Synthes Global AP |
|  | DePuy Synthes Global Advantage |
|  | Implantcast Mutars |
|  | Johnson Delta X-tend monoblock |
|  | Wright Simpliciti |
|  | ZimmerBiomet Nottingham |
|  | ZimmerBiomet Sidus |
|  | ZimmerBiomet TESS |
|  | ZimmerBiomet TM |
